# Hospital-treated infections and subsequent Parkinson’s disease risk: a register-based sibling comparison study

**DOI:** 10.1093/braincomms/fcae098

**Published:** 2024-03-25

**Authors:** Snieguole Vingeliene, Ayako Hiyoshi, Marleen A H Lentjes, Robert J Brummer, Katja Fall, Scott Montgomery

**Affiliations:** Clinical Epidemiology and Biostatistics, School of Medical Sciences, Faculty of Medicine and Health, Örebro University, 703 62 Örebro, Sweden; Clinical Epidemiology and Biostatistics, School of Medical Sciences, Faculty of Medicine and Health, Örebro University, 703 62 Örebro, Sweden; Clinical Epidemiology and Biostatistics, School of Medical Sciences, Faculty of Medicine and Health, Örebro University, 703 62 Örebro, Sweden; Nutrition-Gut-Brain Interactions Research Centre, School of Medical Sciences, Faculty of Medicine and Health, Örebro University, 703 62 Örebro, Sweden; Nutrition-Gut-Brain Interactions Research Centre, School of Medical Sciences, Faculty of Medicine and Health, Örebro University, 703 62 Örebro, Sweden; Clinical Epidemiology and Biostatistics, School of Medical Sciences, Faculty of Medicine and Health, Örebro University, 703 62 Örebro, Sweden; Department of Medical Epidemiology and Biostatistics, Karolinska Institutet, 171 65, Stockholm, Sweden; Clinical Epidemiology and Biostatistics, School of Medical Sciences, Faculty of Medicine and Health, Örebro University, 703 62 Örebro, Sweden; Clinical Epidemiology Division, Department of Medicine, Solna, Karolinska Institutet, 171 77 Stockholm, Sweden; Department of Epidemiology and Public Health, University College London, London WC1E 7HB, UK

**Keywords:** neurodegeneration, cohort study

## Abstract

Serious infections may result in greater risk of Parkinson’s disease. However, high-quality cohort studies focusing on a potential causal role of different types and sites of infection are lacking. Gastrointestinal infections are of a particular interest due to growing evidence implicating gut dysbiosis in Parkinson’s disease aetiology. This population-based cohort study used the Swedish Total Population Register to identify individuals born during 1944–77 and resident in Sweden between 1990 and 2018 (*N* = 3 698 319). Hospital-treated infections at ages 21–30 and 31–40 years were identified from the National Patient Register. Participants were followed to identify Parkinson’s disease diagnoses from age 41 years up to December 31, 2018, when the oldest individual reached 75 years. Cox regression with a sibling comparison design to tackle familial genetic and environmental confounding was used to derive hazard ratios and 95% confidence intervals for each infection site, type, or any infections at ages 21–30 and 31–40 years. During a median follow-up of 15.4 years, 8815 unique Parkinson’s disease diagnoses were accrued, with a crude rate of 17.3 (95% confidence interval 17.0, 17.7) per 100 000 person-years. After controlling for shared familial factors, hospital-treated gastrointestinal and respiratory infections between 21 and 30 years of age were associated with a greater risk of Parkinson’s disease [hazard ratios 1.35 (95% confidence interval: 1.05, 1.75) and 1.45 (95% confidence interval: 1.08, 1.95), respectively]; no association was found for any infections at age 31–40 [hazard ratio 1.05 (95% confidence interval: 0.93, 1.19)]. After adjustment, no statistically significant associations were observed for other sites including genitourinary and skin. These findings suggest that hospital-treated infections of the gastrointestinal tract and lungs, both of which may have an influence on the gut microbiome, by age 30 years may be risk factors for Parkinson’s disease.

## Teaser

Vingeliene *et al*. report that hospital-treated gastrointestinal and respiratory infections at age 21–30 are a risk factor for Parkinson’s disease using sibling comparison design to control for shared familial factors. It is consistent with a role of gastrointestinal microbiome dysbiosis caused by infections in the aetiology of Parkinson’s disease.

## Graphical Abstract

**Graphical Abstract:**
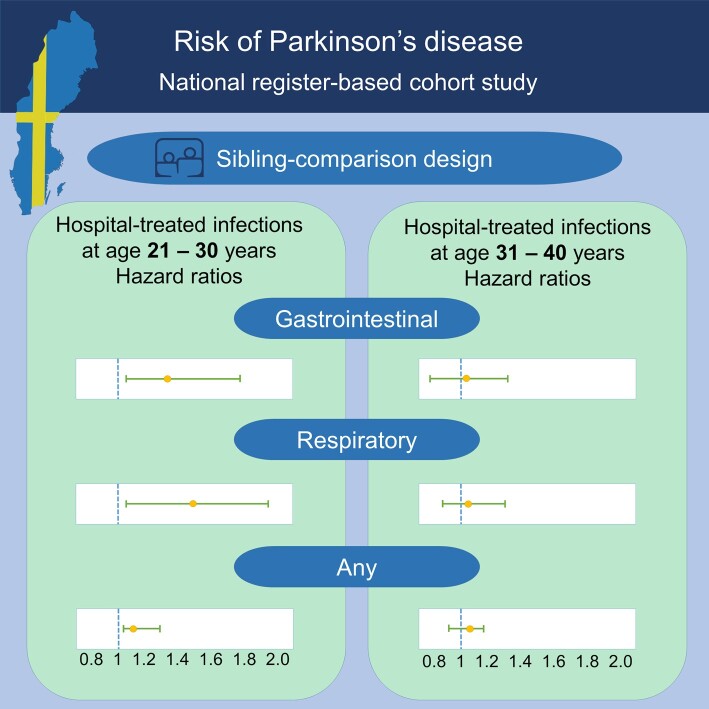

## Introduction

Parkinson’s disease is a neurodegenerative disorder characterized by loss of dopaminergic neurons and abnormal deposits of α-synuclein fibrils, which can lead to motor symptoms including tremor or rigidity, as well as non-motor features including genitourinary, psychiatric, gastrointestinal symptoms and cognitive impairment.^1^ The global burden of Parkinson’s disease has more than doubled from 2.5 million in 1990 to 6.1 million in 2016 and is the fastest growing neurodegenerative disease.^2^ There is no known cure for the disease, so identifying risk factors is of a major importance to better understand its aetiology. The disease seems to originate in the gut in a subset of patients, and there is evidence of gastrointestinal syndromes preceding the diagnosis of Parkinson’s disease.^3^

Some studies indicated a link between bacterial and viral infections and higher risk of Parkinson’s disease.^4^ However, large high-quality cohort studies focusing on a potential causal role for viral infections are lacking. A recent cohort study explored the association of viral infections that occurred between <1 and 15 years prior to Parkinson’s disease.^5^ The association was only statistically significant for infections within 5 years of Parkinson’s disease diagnosis, possibly indicating an increased susceptibility to infection during the prodromal stage of Parkinson’s disease and perhaps earlier diagnosis due to surveillance bias. A Swedish register-based study reported a statistically significant association between any hospital-treated infections and risk of Parkinson’s disease; however, the study did not differentiate between different types and sites of infection.^6^

Gastrointestinal infections are of a particular interest due to the putative gastrointestinal involvement in the development and progression of Parkinson’s disease including impaired synthesis and metabolism of neurotransmitters and a detectable α-synuclein pathology by gut microbiota in individuals with early-stage Parkinson’s disease.^7^ The metagenome is indicative of widespread dysbiosis including due to reduction in species diversity and increased abundance of bacteria capable of eliciting harmful immune responses and inflammation relevant for the Parkinson’s disease risk.^7^ The submucosal plexus and lamina propria in the colon of Parkinson’s disease patients have been shown to contain α-synuclein deposits in the form of Lewy bodies,^8^ and Parkinson’s disease patients display higher gut permeability and lipopolysaccharide (LPS) translocation into the lamina propria^9^ indicating the role of intestinal microbiota and barrier function in the pathogenesis. Braak and colleagues^10^ proposed that the enteric nervous system may represent a pathway between the gut and the CNS, such that environmental influences on the gut could result in the α-synuclein misfolding involved in the aetiology of Parkinson’s disease. Therefore, we hypothesized that serious infections (treated in hospital) of the gastrointestinal tract, but not all other sites, would be associated with a raised risk of Parkinson’s disease. The prodromal stage of Parkinson’s disease is notably variable in duration at the level of the individual, but is on average 10 years in the entire patient group, when some early gastrointestinal symptoms such as constipation,^3,11^ dysphagia, gastroparesis and irritable bowel syndrome without diarrhoea occur.^3^ Therefore, in this study, we considered age at infection to address possible bias due to reverse causation, where prodromal Parkinson’s disease activity may increase infection risk.

The environmental and genetic causes of Parkinson’s disease are incompletely understood, although over 90 distinct genes or loci have been identified since the discovery of the role of accumulation of the insoluble α-synuclein protein.^12^ Genome-wide association studies estimate that up to 36% of Parkinson’s disease risk is due to common genetic variants^13^ and virtually all Parkinson’s disease, including sporadic, may have a genetic component.^14^

In this cohort study, we used Swedish national register data to examine the associations of hospital-treated infections in adulthood (categorized by site, type and age) with subsequent risk of Parkinson’s disease. Sibling comparison analysis was used to address confounding by familial genetic and childhood environmental characteristics, including socioeconomic background.

## Materials and methods

### Study population

This Swedish register-based cohort study includes all individuals born during 1944–77 who were resident in Sweden by 20 years of age between 1990 and 2018 (*N* = 3 698 319). This population was followed up from 41 years of age to the first Parkinson’s disease diagnosis, date of emigration, death or December 31, 2018, whichever occurred first. The oldest individual was ∼75 years of age by the end of follow-up.

The pseudonymized Swedish personal identification number was used to link data from several registers. The Total Population Register provided information on sex, region of residence, dates of birth, immigration, emigration and death.^15^ The National Patient Register (NPR) was initiated in 1964 to register information on in-patient diagnoses and attained full coverage in 1987, with out-patient diagnoses included since 2001.^16^ It was used to identify hospital-treated infections and Parkinson’s disease diagnoses. The Cause of Death Register (CDR) records underlying and contributory causes of death and was also used to identify diagnoses of Parkinson’s disease.^17^ The Multi-Generation Register holds data on biological and adoptive parents for people born after 1931 and residing in Sweden since 1961 and was used to identify biological mothers.^18^ Ethical approval was granted by the Swedish Ethical Review Authority (2019-04755, 2023-03575-02).

### Outcome

A diagnosis of Parkinson’s disease was identified using International Classification of Diseases (ICD) codes ICD-7: 350, ICD-8: 342.00, ICD-9: 332.0 and ICD-10: G20. A validation study indicated that the accuracy calculated as positive predictive value, the proportion of positive register diagnoses being confirmed by the gold standard and sensitivity of Parkinson’s disease diagnoses in the NPR, is 70.8 and 72.7%, respectively.^19^ Reported accuracy and sensitivity in the CDR is 66.7 and 57.1%, respectively.^19^ Date of first Parkinson’s disease diagnosis was defined using a primary or secondary in-patient or out-patient Parkinson’s disease diagnosis from the NPR or an underlying or contributory cause of death from the CDR, whichever occurred first.

### Exposures and potential confounding factors

A diagnosis of primary or secondary in-patient or out-patient hospital-treated infections (not including primary care diagnoses) by site (CNS, gastrointestinal, genitourinary, respiratory and skin), type (bacterial or viral) or any infection was identified using ICD codes (Supplementary Table 1). To examine associations with Parkinson’s disease, hospital-treated infections were identified as occuring during ages 21–30 years and 31–40 years. Both these decades were dichotomized into the person having had or not having had an infection. Due to the data structure, it was not feasible to include ages earlier than 21 years. Sex, geographical region (24 Swedish regions) at the start of follow-up and birth year (continuous variable) were considered as potential confounding factors.

### Statistical analysis

Characteristics of the study participants were summarized using frequencies and percentages by outcome status. Rates of Parkinson’s disease including 95% confidence intervals (CIs) were calculated for each infection site, type (bacterial or viral) or any infection at ages 21–30 and 31–40 years. Hazard ratios (HRs) and 95% CI for each exposure were calculated using Cox proportional hazards regression. To adjust for age, we used age as the underlying time scale. Median follow-up time was calculated using observed length of follow-up. Cohort members who immigrated to Sweden after age 20 years were excluded to ensure no exposure data gaps. Using conventional methods, we fitted two models: age-only adjusted and a model adjusted for all covariates, including two age bands in the same model. To address potential residual confounding shared among siblings, we used a sibling comparison design involving Cox regression analysis with internal stratification by sibling group (those with the same mother). Sex-stratified analysis was conducted using the sibling comparison model. Standard errors were calculated controlling for within-cluster error correlation using mother’s identification number as the cluster variable in all models. Schoenfeld residuals were used to test the proportional hazards assumption, and some violation was found. However, visually assessing survival curves for each exposure, the extent of the violation was minimal and similar results remained after internal stratification. Therefore, we assumed proportionality in the analyses.

Two-sided *P* < 0.05 and 95% CI not including 1.00 were considered statistically significant. All analyses were performed using Stata statistical software version MP 18.0 (StataCorp).

## Results

Among 3 698 319 individuals who were born during 1944–77, a total of 3 165 950 individuals [1 622 122 (51.2%) men and 1 543 828 (48.9%) women] remained after excluding those who emigrated (*n* = 106 818) or died (*n* = 23 203) before the age 41 years, had missing data on geographical region (*n* = 303) or mothers’ identification number (*n* = 398 558).

In the analysis population, 8815 individuals had a Parkinson’s disease diagnosis, at a median age of 61.8 years (IQR, 55.2–67.0; range, 41.0–74.8). Median follow-up was 15.4 years (IQR, 8.0–24.0; range, 0.002–34.0). Compared with those without Parkinson’s disease, those with Parkinson’s disease were more likely to be men and older (Table 1). The proportion of those with and without the diagnosis was similar across the regions (Table 1). The overall rate of Parkinson’s disease was 17.3 (95% CI 17.0, 17.7) per 100 000 person-years (Table 2).

**Table 1:** Characteristics of the study population

	Parkinson’s disease diagnosis	
*N*	Yes (*n* = 8815)	No (*n* = 3 157 135)
Age, years (median, IQR)^a^	61.8 (55.2–67.0)	56.4 (49.0–65.0)
Sex, *n* (%)		
Male	5633 (63.9)	1 616 489 (51.2)
Female	3182 (36.1)	1 540 646 (48.8)
Missing^b^	0	0
Year of birth, *n* (%)		
1944–50	5103 (57.9)	608 100 (19.3)
1951–60	2828 (32.1)	881 974 (27.9)
1961–70	801 (9.1)	1 004 627 (31.8)
1971–77	83 (0.9)	662 434 (21.0)
Missing^b^	0	0
Geographical region, *n* (%)		
Northern Sweden	1357 (15.4)	428 785 (13.6)
South central Sweden	3264 (37.0)	1 225 104 (38.8)
Southern Sweden	4194 (47.6)	1 503 246 (47.6)
Missing^b^	0	303

^a^Age at the diagnosis of Parkinson’s disease or the end of follow-up among those without the disease outcome.

^b^The 303 individuals with missing data were excluded from the main analysis.

**Table 2:** Rate of Parkinson’s disease by infection site and type at ages 21–30 and 31–40 years

Infection, age		*N* Parkinson’s disease/total *N*	Person-years	Rate (95% CI)
**Infection site**				
Total		8815/3 165 950	509.0	17.3 (17.0, 17.7)
Gastrointestinal				
21–30	No	8622/3 087 699	498.1	17.3 (17.0, 17.7)
	Yes	193/78 251	10.9	17.7 (15.3, 20.3)
31–40	No	8664/3 097 887	499.7	17.3 (17.0, 17.7)
	Yes	151/68 063	9.3	16.3 (13.9, 19.1)
Respiratory				
21–30	No	8682/3 104 323	501.7	17.3 (16.9, 17.7)
	Yes	133/61 627	7.3	18.3 (15.4, 21.7)
31–40	No	8637/3 048 836	496.6	17.4 (17.0, 17.8)
	Yes	178/117 114	12.4	14.3 (12.4, 16.6)
CNS				
21–30	No	8794/3 157 898	507.7	17.3 (17.0, 17.7)
	Yes	21/8052	1.2	17.0 (11.1, 26.1)
31–40	No	8794/3 156 964	507.7	17.3 (17.0, 17.7)
	Yes	21/8986	1.3	16.5 (10.8, 25.4)
Genitourinary				
21–30	No	8789/3 148 650	507.0	17.3 (17.0, 17.7)
	Yes	26/17 300	2.0	12.8 (8.7, 18.8)
31–40	No	8787/3 143 371	506.6	17.3 (17.0, 17.7)
	Yes	28/22 579	2.3	12.0 (8.3, 17.4)
Skin				
21–30	No	8758/3 135 950	505.5	17.3 (17.0, 17.7)
	Yes	57/30 000	3.4	16.5 (12.8, 21.4)
31–40	No	8756/3 112 285	504.1	17.4 (17.0, 17.7)
	Yes	59/53 665	4.9	12.1 (9.4, 15.6)
**Infection type**				
Bacterial				
21–30	No	8242/2 898 573	466.1	17.7 (17.3, 18.1)
	Yes	573/267 377	42.9	13.4 (12.3, 14.5)
31–40	No	8312/2 879 728	474.3	17.5 (17.2, 17.9)
	Yes	503/286 222	34.7	14.5 (13.3, 15.8)
Viral				
21–30	No	8691/3 100 213	501.5	17.3 (17.0, 17.7)
	Yes	124/65 737	7.5	16.6 (13.9, 19.8)
31–40	No	8662/3 045 344	497.5	17.4 (17.1, 17.8)
	Yes	153/120 606	11.5	13.3 (11.3, 15.6)
**Any infection**				
21–30	No	7883/2 752 106	446.6	17.7 (17.3, 18.0)
	Yes	932/413 844	62.3	15.0 (14.0, 15.9)
31–40	No	7983/2 683 085	451.5	17.7 (17.3, 18.1)
	Yes	832/482 865	57.5	14.5 (13.5, 15.5)

Rate: per 100 000 person-years.

In the analysis by infection site (Table 3), those with gastrointestinal infection at ages 21–30 years had a greater risk of being diagnosed with Parkinson’s disease [model 1 HR 1.23 (1.06, 1.41)] compared with people who did not have this type of infection between these ages. The association remained statistically significant after covariate adjustment and use of the sibling comparison method [HRs 1.21 (1.05, 1.40) and 1.35 (1.05, 1.75), respectively]. There was no association with Parkinson’s disease for gastrointestinal infections between ages 31 and 40 years. Respiratory infections at 21–30 years of age were associated with greater risk of being diagnosed with Parkinson’s disease in sibling comparison model [HR 1.45 (1.08, 1.95)]. There was a lower magnitude association between respiratory infections at ages 31–40 years and Parkinson’s disease, but this association was attenuated and became statistically non-significant in the sibling comparison analysis [HR 1.04 (0.81, 1.34)]. We observed no greater risk of being diagnosed with Parkinson’s disease among those who had CNS, genitourinary or skin infections in either of the age strata.

**Table 3:** Association between hospital-treated infections at ages 21–30 and 31–40 years and risk of Parkinson’s disease

Infection, age	Cox proportional hazards regression, HR (95% CI)					
	Conventional				Stratified^a^	
	Model 1^b^	*P*	Model 2^c^	*P*	Model 3^c^	*P*
**Infection site**						
Gastrointestinal						
21–30	1.23 (1.06, 1.41)	0.005	1.21 (1.05, 1.40)	0.01	1.35 (1.05, 1.75)	0.02
31–40	1.05 (0.90, 1.23)	0.54	1.03 (0.88, 1.21)	0.70	1.02 (0.76, 1.37)	0.88
Respiratory						
21–30	1.35 (1.13, 1.60)	0.001	1.36 (1.14, 1.61)	<0.001	1.45 (1.08, 1.95)	0.01
31–40	1.18 (1.02, 1.37)	0.03	1.18 (1.01, 1.37)	0.03	1.04 (0.81, 1.34)	0.76
CNS						
21–30	1.11 (0.72, 1.71)	0.63	1.14 (0.74, 1.75)	0.55	1.73 (0.82, 3.67)	0.15
31–40	1.04 (0.68, 1.59)	0.87	1.04 (0.68, 1.60)	0.85	0.68 (0.33, 1.38)	0.29
Genitourinary						
21–30	0.83 (0.57, 1.22)	0.35	1.00 (0.68, 1.48)	0.99	1.39 (0.75, 2.59)	0.30
31–40	0.94 (0.65, 1.36)	0.74	1.09 (0.75, 1.59)	0.64	1.63 (0.95, 2.80)	0.08
Skin						
21–30	1.17 (0.90, 1.51)	0.25	1.11 (0.85, 1.44)	0.45	0.92 (0.53, 1.57)	0.75
31–40	1.15 (0.89, 1.49)	0.28	1.08 (0.84, 1.40)	0.54	1.27 (0.81, 1.98)	0.30
**Infection type**						
Bacterial						
21–30	0.77 (0.71, 0.84)	<0.001	1.01 (0.92, 1.10)	0.87	1.07 (0.93, 1.24)	0.35
31–40	0.93 (0.85, 1.02)	0.14	1.15 (1.05, 1.26)	0.004	1.13 (0.97, 1.33)	0.11
Viral						
21–30	1.21 (1.01, 1.44)	0.04	1.21 (1.01, 1.44)	0.04	1.03 (0.74, 1.42)	0.88
31–40	1.22 (1.04, 1.43)	0.02	1.20 (1.02, 1.41)	0.03	1.15 (0.86, 1.54)	0.36
**Any infection**						
21–30	0.90 (0.84, 0.96)	0.002	1.09 (1.02, 1.17)	0.02	1.15 (1.02, 1.30)	0.02
31–40	0.97 (0.90, 1.04)	0.38	1.09 (1.02, 1.18)	0.01	1.05 (0.93, 1.19)	0.44

HR, hazard ratio.

^a^Sibling comparison analysis where mother’s identification number was used as the stratification variable.

^b^Adjusted for age using age as the underlying time scale.

^c^Adjusted for sex (male, female), birth year (continuous) and 24 Swedish regions of residence. All models were adjusted for age at infection by the use of age as the time scale.

Although some associations by infection type (bacterial or viral) were statistically significant in conventional models, none of the associations remained statistically significant using the sibling comparison design (Table 3).

After controlling for unmeasured shared familial factors using the sibling comparison design, only any infection at 21–30 years remained statistically significantly associated with Parkinson’s disease [HR 1.15 (1.02, 1.30); Table 3].

In sex-stratified analysis, men who had a gastrointestinal infection at ages 21–30 years were at a greater risk of being diagnosed with Parkinson’s disease than men who did not have this type of infection at these ages [HR 1.51 (1.01, 2.26)]. The HR for an association between respiratory infection at ages 21–30 years and risk of Parkinson’s disease among men was similar to the HR in the main analysis but no longer statistically significant [HR 1.51 (0.89, 2.56)]. Accurate estimation for an association with other infections was not possible due to lack of power, producing wide CIs (Supplementary Table 2). Among women, none of the estimates for Parkinson’s disease risk by infection site or type were statistically significant (Supplementary Table 2).

## Discussion

In this nationwide register-based cohort study, we found that hospital-treated infection in early adulthood was associated with a raised risk of subsequent Parkinson’s disease. After adjustment, the associations were limited to gastrointestinal and respiratory infections, occurring at ages 21–30 years and not at 31–40 years. The use of sibling comparison models adjusted for familial genetic and childhood environmental characteristics shared among siblings. We believe that these findings further implicate the gut, possibly including alterations to the microbiome, in the aetiology of Parkinson’s disease.

There have been few other high-quality studies investigating infections and risk of Parkinson’s disease.^4^ The current evidence comes from case–control studies with relatively small sample sizes and exposure diagnosis close to the date of outcome diagnosis.^20^ A recent longitudinal study reported an association between influenza and risk of Parkinson’s disease but only within 5 years prior to the diagnosis of Parkinson’s disease.^5^ The prodromal period in Parkinson’s disease may start as early as 10–20 years before the diagnosis^11^; therefore, infections a few years prior to the diagnosis may indicate increased susceptibility to pathogens due to Parkinson’s disease activity rather than the infection increasing Parkinson’s disease risk. A recent Swedish study reported an association between any infection before 40 years of age and risk of Parkinson’s disease with a 10-year lag time, but did not investigate by site or type of infection, or use the sibling comparison method as a method to further reduce residual confounding.^6^

By examining infections between 21–30 and 31–40 years, we sought to assess the likelihood that the infections were a cause of Parkinson’s disease or a consequence of the prodromal period of Parkinson’s disease. While we found an elevated risk of Parkinson’s disease among those who had gastrointestinal and respiratory infections between 21 and 30 years of age, no significant associations were observed for ages 31–40 years, making it less likely that infections were due to prodromal Parkinson’s disease. This study included only hospital-treated infections in in-patient or out-patient settings, indicating higher clinical severity and accuracy in recording the site of infection. The association of all types of infection between ages 21–30 years and Parkinson’s disease risk was driven by gastrointestinal and respiratory infections at these ages. The lack of association for infections divided by viral or bacterial is likely because site of infection was not considered in the planned analysis.

Due to the important genetic influence on Parkinson’s disease risk,^13^ we used sibling comparison models, where the risk of outcome is compared between siblings. In addition to adjusting for the genetic characteristics shared between siblings, this method adjusts effectively for shared family background characteristics such as parental socioeconomic circumstances, so there is no reason to additionally adjust for measures of parental social position that would be more likely to result in residual confounding. Some of the statistically significant associations produced by conventional Cox regression models were attenuated and became statistically non-significant in the sibling comparison models, indicating that the conventional analysis may have been the result of confounding by familial characteristics. The associations of gastrointestinal and respiratory infections at ages 21–30 with Parkinson’s disease remained in the results of sibling comparison analysis, indicating robustness of the association independent of genetic and socioeconomic characteristics.

We hypothesized that serious gastrointestinal infections in early adulthood might increase the risk of Parkinson’s disease as a consequence of the disruption of the gut microbiome, the resulting dysbiosis and its influence through gut–brain pathways involving the enteric nervous system and the autonomous nervous system in accordance with Braak’s hypothesis of Parkinson’s disease pathogenesis.^10^  *Escherichia coli* and *Klebsiella* species that are elevated in Parkinson’s disease^7^ produce amyloidogenic protein, curli, which has been demonstrated to be involved in α-synuclein pathology, inflammation and neurodegeneration in the brain.^21^ A large-scale metagenomics study of Parkinson’s disease patients identified 11 bacterial species of Gram-negative bacteria with LPSs, capable of eliciting strong immune reaction and inflammation via Toll-like receptor 4 signalling pathway,^22,23^ whereas *Prevotella copri* that can inhibit this activation was reduced.^7^ In addition, elevated levels of gene families and pathways involved in production of LPS and highly immune stimulatory bacterial lipoprotein were also detected. Early Parkinson’s disease symptoms such as constipation, compromised gut barrier and inflammation may be driven by reduction in bacterial species involved in short-chain fatty acid production, such as butyrate, as well as in synthesis and metabolism of serotonin, which affects gut motility.^7^

The only other site of serious infections linked with raised Parkinson’s disease risk was respiratory, and this could potentially also be because of an influence on the gut microbiome. A systematic review of respiratory infections, predominantly of studies examining adults, found a decrease in diversity of the gut microbiome following infection^24^ compared with people who were uninfected. For example, viral influenza has been associated with *E. coli* overgrowth likely due to wider systemic immunological consequences of the infection,^25^ as there are immune and circulatory system pathways between the lungs and gut.^26^ An influence of respiratory infections on the microbiome is a plausible explanation of the increase in Parkinson’s disease risk associated with serious respiratory infections during young adulthood, but an alternative explanation is that respiratory infections also influence Parkinson’s disease risk through an olfactory pathway.^10,27^ It is likely that the infections themselves played the most important role rather than antibiotic treatment in explaining the association of gastrointestinal and respiratory infections with Parkinson’s disease: bacterial infections at other sites, not associated with Parkinson’s disease, would also be treated using antibiotics.

Study strengths include a large, nationwide sample, with site- and age-specific exposure measures, and complete follow-up due to linkage between national registers. The registers provided prospectively recorded diagnoses of infections and Parkinson’s disease, further minimizing the risk of selection and measurement bias that would be likely in studies dependent on retrospective data collection. There are also some potential limitations. We could not examine hospital-treated infections before age 21 years, as register coverage was insufficient for those ages in individuals with sufficient follow-up time to identify Parkinson’s disease diagnoses. Misclassification of infection status at age 21–30 years among individuals born in earlier years, prior to the full coverage of national registers, is unlikely to have created a spurious positive association, as non-differential misclassification would bias results towards the null, assuming the absence of other biases. Multiple types of infection were examined, increasing the risk of a chance finding due to multiple testing. Our *a priori* hypothesis was that gastrointestinal infections represent a raised risk for Parkinson’s disease. While the association with respiratory infections could be a chance finding identified through multiple testing, this finding is also consistent with theories of Parkinson’s disease aetiology. The maximum age at diagnosis in our study was 74.8 years. While diagnoses at older ages were not captured, this may have benefits for sensitivity and accuracy. The proportion of gold standard positive Parkinson’s disease diagnoses identified in the registers is highest up to age 75 years (81.6% at ≤60 years; 78.6% at 61–75 years; and 42.9% at ≥76 years). Similarly, the accuracy of Parkinson’s disease diagnoses is higher among younger individuals (85.0% at ≤64 years; 78.6% at 65–70 years; and 60.5% at ≥71 years).^19^ As out-patient diagnoses were only available from 2001 onwards, some earlier Parkinson’s disease diagnoses may not have been identified among those who did not survive beyond this time. A proportion would have been identified from CDR data, but the quality of diagnoses from death certificates is often inferior to hospital diagnoses. Some diagnoses of Parkinson’s disease will have been missed, but this is likely to have resulted in non-differential bias, possibly driving the observed associations towards the null. Importantly, we did not measure disruption of the gut microbiome and gut–brain signalling directly, only using infection as a proxy marker of exposure relevant to gut–brain signalling.

In conclusion, hospital-treated infections that may have gastrointestinal consequences by age 30 years are associated with an elevated risk of Parkinson’s disease. This is consistent with a role for gastrointestinal microbiome dysbiosis (or other influences relevant to gut–brain signalling) caused by the infections in the aetiology of Parkinson’s disease.

## Supplementary Material

fcae098_Supplementary_Data

## Data Availability

The data used in this study cannot be shared publicly due to regulations under Swedish law. Inquiries about the data used in the present study can be addressed to the corresponding author. Researchers who are interested in Swedish register data can refer to https://www.registerforskning.se/en/.
