# Supplementary material for: Hospital-treated infections and subsequent Parkinson’s disease risk: a register-based sibling comparison study
Source: Brain Commun. 2024 Mar 25;6(2):fcae098. doi: 10.1093/braincomms/fcae098 (PMC10984571; doi:10.1093/braincomms/fcae098)
Supplement: fcae098_Supplementary_Data [file fcae098_supplementary_data.docx]

**Supplementary material**

**Supplementary Table 1. ICD codes for infections used in this study.**

|  | **ICD-8 (1969-1986)** | **ICD-9 (1987-1996)** | **ICD-10 (1997-2018)** |
| --- | --- | --- | --- |
| Infectious mononucleosis | 075 | 075 | B27 |
| Pneumonia | 480-486 | 480-486 | J12-J18, P23 |
| Central nervous system | 013,062,063,064,065,  066,071,094,292,320,  323,324, 390,474,  040-046 | 013,062,063,064,071,094,320,323,326,392,  045-049 | A17,A80-A89,G00,G01,  G02,G04,G05,I02 |
| Gastrointestinal | 014,123,127,129,540,  567,000-009 | 014,123,127,129,540,  567,000-009 | A00-A09,B71,B81,B82,  K35,K65,K67 |
| Genitourinary | 016,590,595,597 | 016,590,595,597 | N30,N34,O23 |
| Respiratory | 010,011,012,033,034,  075,115,116,490,501,  503,510, 460-466,470-474,480-486 | 010,011,012,033,034,  075,115,116,473,475,  487,490,510, 460-466,480-486 | A15,A16,A37,A38,B27,  B39,B40,B41,B42,B44,  B59, J00-J06,J10,  J12-J18,J20-J22,J32,J36,  J40-J42,J86,P23 |
| Skin | 110,111,050-057,  680-686 | 110,111,050-057,  680-686 | B00-B09,B35,B36,B43,  L00-L08 |
| Bacterial | 000,001,002,003,004,005,073,076,080,081,082,  083,320,362,380,381,  382,383,421,461,481,  482,501,510,567,590, 595,597,612,613,614,616,620,622,630,635,670,  678,680,681,682,684,  710,720,010-019,020-027,030-039, 090-099,100-104,390-392 | 001,002,003,004,005,073,076,077,078,079,080,081, 082,083,320,381,  382,383,421,461,475,481,482,510, 567,590,595,  597,670,730,010-018,  020-027,030-041,  090-099,100-104,  390-392,614-616,  680-686 | A00,A01,A02,A03,A04,A05,A15-A19,A20-A28, A30-A49,A50-A58,A65-A69,A70-A74, A75-A79,  B95,B96,G00,G01,H60,  H70,I00-I02,I33,J01, J13,J14,J15,J36,J86,K65,  L00-L08,M00,M86,  N30,N34, N70-N77,  O23,O85,O86,P36 |
| Viral | 075,360,420,422,460,464,465,466,480,  040-046,050-057,  060-068,070-079,  470-474 | 070,071,072,074,075,077,078,079,372,420,422,  460,464, 465,466,480,  487,647,711,045-049,  050-057,060-066 | A08,A60,A80-A89,A90-A99,B00-B09,B15-B19, B20-B24,B25-B34,B27,  B97,B99,H10,I30,I40,J00,J04, J05,J06,J10,J12,  J20,J21,O98,P35,Z21 |
| Other | 084,085,086,087,088,089,112,113,114,117,120,  121,122, 124, 125,126,  128,130, 131,132,133,  134,135,136,363,610,611, 615,732,763,Y41 | 084,085,086,087,088,112,113,114,117,118,120,121,122, 124,125,126,  128,130,131,132,133,  134,135,136,370,675, 771,137-139 | V02,B37,B38,B45,B46,B47,B48,B49,B50-B58,  B60-B64, B65-B70,  B72-B80,B83,B85-B89,  B90-B94,H16,H32, M01,  M02,M03,O91,P37,P38,P39,Z22, A59,A63 ,A64 |

**Supplementary Table 2. Association between hospital-treated infections at the age of 21-30 and 31-40 years and risk of subsequent Parkinson’s disease among individuals born in 1944 - 1978, using a sibling comparison analysis in men and women.**

|  | **Cox proportional hazards regression, HR (95% CI)** | | | |
| --- | --- | --- | --- | --- |
|  |  |  |  |  |
| **Infection, age** | **Men** | **P** | **Women** | **P** |
| **Any infection** |  |  |  |  |
| 21 – 30 | 1.50 (1.14, 1.96) | 0.003 | 0.98 (0.79, 1.20) | 0.82 |
| 31 – 40 | 1.21 (0.95, 1.53) | 0.13 | 1.03 (0.83, 1.27) | 0.82 |
| **Infection type** |  |  |  |  |
| Bacterial |  |  |  |  |
| 21 – 30 | 1.38 (0.88, 2.15) | 0.16 | 1.01 (0.81, 1.26) | 0.93 |
| 31 – 40 | 1.59 (1.11, 2.28) | 0.01 | 1.05 (0.82, 1.35) | 0.69 |
| Viral |  |  |  |  |
| 21 – 30 | 1.26 (0.70, 2.29) | 0.44 | 0.76 (0.36, 1.59) | 0.46 |
| 31 – 40 | 1.31 (0.84, 2.05) | 0.24 | 0.90 (0.46, 1.77) | 0.76 |
| **Infection site** |  |  |  |  |
| Central nervous system |  |  |  |  |
| 21 – 30 | 4.6 (1.04, 20.74) | 0.04 | 1.58 (0.25, 10.09) | 0.63 |
| 31 – 40 | 1.11 (0.38, 3.24) | 0.86 | 0.29 (0.03, 2.53) | 0.27 |
| Gastrointestinal |  |  |  |  |
| 21 – 30 | 1.51 (1.01, 2.26) | 0.05 | 1.01 (0.56, 1.81) | 0.98 |
| 31 – 40 | 1.05 (0.64, 1.70) | 0.85 | 1.68 (0.96, 2.96) | 0.07 |
| Genitourinary |  |  |  |  |
| 21 – 30 | 3.85 (0.61, 24.22) | 0.15 | 2.33 (0.82, 6.65) | 0.11 |
| 31 – 40 | 9.18 (1.05, 80.48) | 0.05 | 0.88 (0.34, 2.30) | 0.80 |
| Respiratory |  |  |  |  |
| 21 – 30 | 1.51 (0.89, 2.56) | 0.13 | 1.02 (0.57, 1.83) | 0.94 |
| 31 – 40 | 0.91 (0.60, 1.37) | 0.64 | 1.43 (0.85, 2.43) | 0.18 |
| Skin |  |  |  |  |
| 21 – 30 | 1.37 (0.63, 3.00) | 0.43 | 0.32 (0.07, 1.49) | 0.15 |
| 31 – 40 | 1.95 (0.96, 3.95) | 0.07 | 0.43 (0.12, 1.54) | 0.20 |

HR, hazard ratio.

Data were stratified by sex (male, female), and estimates are obtained from sibling-comparison approach with adjustment for birthyear (continuous) and 24 Swedish regions of residence. All models were adjusted for age at infection by the use of age as the time scale.
